# Analysing the Interaction between Microscopic Deformation, Microstructure and Void Evolution of Near-α Titanium Alloys during Non-Superplastic Hot Deformation by an Integrated Crystal Plasticity Finite Element Model

**DOI:** 10.3390/ma15010294

**Published:** 2021-12-31

**Authors:** Jie Zhao, Kehuan Wang, Liangxing Lv, Liliang Wang, Denis J. Politis, Gang Liu

**Affiliations:** 1 National Key Laboratory for Precision Hot Processing of Metals, Harbin Institute of Technology, Harbin 150001, China; zhaojiemse@163.com (J.Z.); wangkehuan@hit.edu.cn (K.W.); 2 Institute of High Pressure Fluid Forming, Harbin Institute of Technology, Harbin 150001, China; 3 The Space Environment Simulation and Research Infrastructure (SESRI), Harbin Institute of Technology, Harbin 150001, China; lvliangxing@hit.edu.cn; 4 Department of Mechanical Engineering, Imperial College London, Exhibition Road, London SW7 2AZ, UK; liliang.wang@imperial.ac.uk (L.W.); denis.politis06@imperial.ac.uk (D.J.P.); 5 Department of Mechanical and Manufacturing Engineering, University of Cyprus, Nicosia 1678, Cyprus

**Keywords:** crystal plasticity finite element model, near-α titanium alloys, non-superplastic hot deformation, microscopic deformation

## Abstract

High-efficiency and low-cost hot forming technologies for titanium alloys have been developed for producing complex-shaped, thin-walled tubular components under non-superplastic forming conditions. Under these forming conditions, there exist complex and highly integrated material evolution processes including microscopic heterogeneous deformation, microstructure evolution and damage behaviour. This paper presents an integrated crystal plasticity finite element model of near-α titanium alloys during non-superplastic hot deformation conditions considering grain boundary sliding (GBS), dynamic recrystallisation (DRX), as well as void evolution. The polycrystalline model of a near-α TA15 titanium alloy was established, containing α phase, β phase and grain boundary (GB) regions, in which the GB region was a visualised representation of GBS. The quantitative strength ratio between the GB regions and α phase was calculated according to the Zener–Holloman parameter *Z* and grain size, which determined the microscopic deformation behaviour. There were found to be two high microscopic strain regions in the α phase: intragranular deformation bands through the most favourable slipping and near the GBs through multiple slipping, which promoted continuous and discontinuous DRX, respectively. With the decrease in parameter *Z* or grain size, the activated dislocations accommodating GBS were found to no longer pile up inside the grain, but instead travel across the grain interior. Finally, methods to improve the macroscopic plastic formability were proposed for the difficult-to-form titanium alloys experiencing non-superplastic hot deformation.

## 1. Introduction

Near-α titanium alloys are widely used in the aerospace industry due to their outstanding properties, including their superior specific strength and excellent corrosion resistance [1]. However, due to the brittle nature of HCP metals in low temperature, most titanium alloys are processed by means of thermo-mechanical processing. High-efficiency and low-cost hot forming technologies for titanium alloys have been developed for producing complex-shaped, thin-walled tubular components, such as hot gas pressure forming with the unique characteristics of the relatively low forming temperature and high strain rate, i.e., the non-superplastic region [2]. The complex interactions between microscopic heterogeneous deformation, microstructure evolution and damage behaviour result in titanium alloys being extremely difficult to control in terms of shape and performance characteristics simultaneously [3]. A thorough understanding of microscopic deformation of titanium alloys during non-superplastic hot deformation can help guide the forming process to control the formability and performance of the components.

The hot microscopic deformation behaviour of titanium alloys can be divided into three aspects: the heterogeneous deformation (stress and strain distribution caused by the non-uniform deformation mechanism), the microstructure evolution (including texture evolution, dislocation density evolution, dynamic recovery, dynamic recrystallisation and phase transformation) and the damage behaviour (consisting of the void and microcrack evolution). To address the complexity of coupling these three aspects, the crystal plasticity-based simulation framework is an ideal alternative tool for the study of microscopic strain heterogeneity [4], microstructure evolution [5] and ductile fracture behaviour [6]. However, due to the complex deformation mechanisms, microstructure evolution and difficult-to-obtain material model parameters, there has been limited work related to the crystal plasticity modelling of titanium alloys during non-superplastic hot deformation conditions.

The common hot deformation mechanisms of titanium alloys include dislocation motion, grain boundary sliding (GBS), twinning and diffusion where multiple mechanisms may coexist. High deformation temperature and high Al content limit the occurrence of twinning [7], while diffusion is the main hot deformation mechanism at high temperature and low strain rate, such as creep [8]. Therefore, the main hot deformation mechanisms of titanium alloys during non-superplastic hot deformation are dislocation motion and GBS. However, in most existing crystal plasticity models of titanium alloys, dislocation motion serves as the unique hot deformation mechanism. The temperature-dependent evolution of the anisotropy and asymmetry of the α-Ti tube in thermomechanical working has been determined through a crystal plasticity-based numerical simulation. The anisotropy and asymmetry in terms of flow stress are significantly reduced at elevated temperatures due to the decrease in twinning, which depends on the decrease in the critical resolved shear stress (CRSS) value of pyramidal 〈c+a〉 slip with temperature [9]. As for the heterogeneous deformation of two-phase titanium alloys with a colony structure during hot deformation, an explicit crystal plasticity model has been used to investigate the hot compression of the single colony. The heterogeneous deformation can be attributed to the geometrical orientation of the testing colony as well as Hall–Petch strengthening between constituent phases [10]. A crystal plasticity model was developed to analyse the microscopic deformation of a near-α titanium alloy with an equiaxed structure during hot deformation, in which the localised stress concentration tends to spread at the parts of hard grains adjacent to soft grains, yet the distributions of strain manifest in a sequential fashion [11]. To consider the GBS mechanism in the crystal plasticity model, Li et al. introduced a scaling factor to control the dislocation density (viz., strength) of the grain boundary (GB) region to be lower than the grain core and described the enhanced contribution of GBS to plastic deformation during hot deformation in the β-single phase region of a near-α titanium alloy [12]. Therefore, unlike the dislocation motion model, an optimized physics-based GBS crystal plasticity model needs to be developed to analyse the compatible deformation of two-phase polycrystalline titanium alloys during hot deformation.

The heterogeneous deformation of grains caused by the non-uniform deformation mechanism determines the microstructure evolution such as texture evolution, dislocation density evolution, dynamic recovery (DRV), dynamic recrystallisation (DRX) and phase transformation. The changes of texture, dislocation density, grain morphology and phase fraction in turn alters the subsequent heterogeneous deformation. The prediction of microstructure evolutions during thermo-mechanical processes through crystal plasticity modelling is still challenging. Li et al. established a 3D coupling model of crystal plasticity and cellular automata (CA), in which the heterogeneous deformation and the non-uniform distribution of the dislocation density were calculated through a crystal plasticity model, and the morphological evolution of DRX was calculated with CA through a semi-probabilistic switch rule. The coupled effect of the heterogeneous deformation, mechanical response and DRX evolution during the isothermal compression in the β-single phase region of a near-α titanium alloy was well-captured [12]. Subsequently, Li et al. coupled the Monte Carlo and crystal plasticity model to capture the thermal interface grooving and deformation anisotropy during the isothermal compression of the IMI834 titanium alloy with a lamellar colony [13]. In addition, the integrated crystal plasticity and phase field model is another powerful tool for the interaction between microscopic deformation and microstructure evolution [5]. However, as for the non-superplastic hot deformation of titanium alloys, the grain refinement caused by DRX can promote the GBS mechanism [14]. The interaction between GBS and DRX has not been fully considered in the existing crystal plasticity-based framework of titanium alloy non-superplastic hot deformation.

The formability of titanium alloys during non-superplastic hot deformation is limited. The damage behaviour is governed by the mechanisms of void nucleation, growth and coalescence, and the voids tend to nucleate on the phase boundaries for the two-phase titanium alloys [15]. The microscopic crack nucleation point can be predicted by the distribution of the microscopic stress, strain and dislocation density [16]. A crystal plasticity-based crack nucleation model was developed for two-phase titanium alloys incorporating the room temperature creep phenomenon. The soft grains with preferential orientation undergo an obvious plastic strain, while the local stress concentration occurs at the GBs of hard grains, leading to microscopic crack initiation [17]. Based on the crystal plasticity-based simulation of two-phase titanium alloys, the phase boundary inclination has a very strong impact on the void growth, and the choice of Burgers orientation relationship variant affects the void growth in moderate triaxialities [15]. Moreover, the damage behaviour of metal materials during hot deformation is closely related to microstructure evolution. For example, DRX can relieve the local stress concentration and refine the grain size, which decreases the deformation heterogeneity and improves the ductility of the material [18,19]. However, void evolution, considering the complicated microscopic compatible deformation of two-phase polycrystalline titanium alloys during non-superplastic hot deformation, has not been analysed well by crystal plasticity modelling.

**Figure 1:**
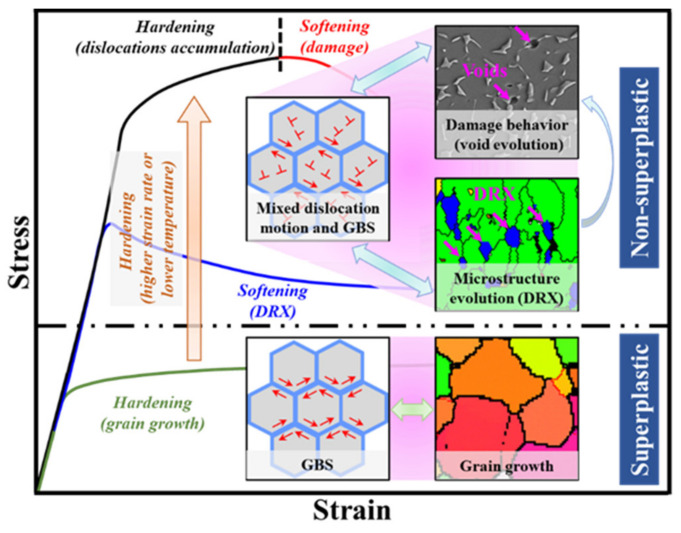
Interaction relationship of the microscopic heterogeneous deformation, the microstructure evolution and the damage behaviour of titanium alloys during hot deformation.

This paper aims to develop an integrated crystal plasticity model of near-α titanium alloys during non-superplastic hot deformation, considering the deformation mechanisms (dislocation motion and GBS), microstructure evolution (dislocation density, DRV and DRX) and void evolution. The coupling effects of microscopic heterogeneous deformation, microstructure evolution and void evolution, as shown in Figure 1, are analysed. Moreover, the methods to improve the macroscopic plastic formability of titanium alloys during non-superplastic hot deformation are discussed.

## 2. Integrated Crystal Plasticity Finite Element Model

### 2.1. Material Characterisation and Polycrystalline Model

**Figure 2:**
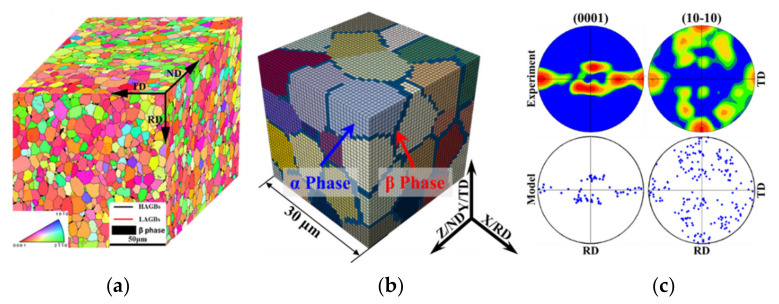
Microstructure characterisation and polycrystalline model of the undeformed near-α TA15 sheet. (**a**) Inverse pole figures [11,20]; (**b**) polycrystalline model; (**c**) comparison of grain orientations in experiment and simulation.

A near-α TA15 titanium alloy rolled sheet with a chemical composition of Ti-6.5Al-2Zr-1Mo-1V (in wt%) and a thickness of 2 mm was utilised in this work. The microstructure of TA15 samples was characterised by EBSD performed on a FEG LEO 1530 SEM with a scan size of 0.3 μm. The inverse pole figure of the undeformed TA15 sheet is depicted in Figure 2a, in which the colour code represents the α grain orientation, black lines represent the high angle grain boundaries (HAGBs, θ > 15°) and red lines represent the low angle grain boundaries (LAGBs, 2° < θ < 15°). The average α grain size of the TA15 sheet was 9.72 μm. The TA15 titanium alloy sheet underwent a recrystallised annealing process, and the low fraction of LAGBs (~14.3%) indicated that the sheet contained few deformed structures. The β phase was distributed at the GBs of the α grains in a connected network, and the volume fraction was approximately 10%, as shown in the black region in Figure 2a.

The polycrystalline model, based on the equiaxed microstructure of the TA15 titanium alloy, is shown in Figure 2b, which was realised by the hexahedral element in ABAQUS software. The three-dimensional size of the polycrystalline model was 0.03 × 0.03 × 0.03 mm^3^, which contained 50 α grains. The β phase was distributed at the GBs of the equiaxed α grains. The initial grain orientations of the polycrystalline model were extracted from the EBSD data of the undeformed TA15 sheet. Fifty randomly selected initial orientations were distributed into the polycrystalline model randomly, which was able to properly characterise the initial texture of the TA15 sheet [20]. The comparison of the grain orientations in the simulation and the experimental results is shown in Figure 2c, in which RD is the rolling direction and TD is the transverse direction of the sheet.

### 2.2. Constitutive Model of Titanium Alloy during Non-Superplastic Hot Deformation

#### 2.2.1. Kinetic Equation of Dislocation Motion

During the hot deformation process of metals, dislocations move under the coaction of external force and thermal activation, including not only dislocation slipping but also diffusion-controlled dislocation climbing. In the kinetic equation of dislocation motion, the activation energy of deformation is usually used to express the diffusion-controlled dislocation motion [21]. In addition, according to the classic Orowan equation, the shear strain rate of dislocations is related to the mobile dislocation density. Therefore, this paper considered the influence of deformation temperature, strain rate, deformation activation energy and mobile dislocation density on the dislocation motion simultaneously, and established the kinetic equation of dislocation motion, which is given as:

(1) $${\dot{\gamma }}^{\alpha }={\left[c_{1}\rho_{m}^{\alpha }exp\left(-\frac{Q_{Act}}{RT}\right)\left|\frac{\tau^{\alpha }}{\tau_{pass}^{\alpha }}\right|\right]}^{1/m}sign\left(\tau^{\alpha }\right)$$

where ${\dot{\gamma }}^{\alpha }$ is the shear strain rate,$\;c_{1}$ is a fitting constant, $\rho_{m}^{\alpha }$ is the mobile dislocation density, $Q_{Act}$ is the activation energy of deformation, R is the gas constant, T is the deformation temperature, $\tau_{pass}^{\alpha }$ is the dislocation motion resistance and m is the strain rate sensitivity exponent.

The dislocation motion resistance usually adopts the Taylor hardening model, as shown in Equation (2). The dislocation density in the hardening model is reflected by the relative dislocation density proposed by Lin et al. [22], as shown in Equation (3). The adoption of the relative dislocation density is conducive to the numerical realisation and the parameter solution of the constitutive model.

(2) $$\tau_{pass}^{\alpha }=\overset{N}{\underset{\beta =1}{\sum }}c_{2}{\bar{\rho }}^{\beta }{}^{0.5}$$

(3) $${\bar{\rho }}^{\alpha }=\rho^{\alpha }/\rho_{0}^{\alpha }$$

where $c_{2}$ is a fitting constant, ${\bar{\rho }}^{\beta }$ is the relative dislocation density of the slip system β, $\rho_{0}^{\alpha }$ is the initial dislocation density of the slip system α and $\rho^{\alpha }$ is the dislocation density of the slip system α after deformation.

During the hot deformation process, mobile dislocations will rapidly multiply and annihilate, and will also become immobile dislocations due to the pinning effect of the second phase or forest dislocations. Due to the complexity of the evolution of mobile dislocations, the simplified proportional relationship between the mobile dislocation density and the overall dislocation density is usually established directly in the constitutive model [23]. Therefore, this paper established a simplified expression of mobile dislocation density with deformation temperature, strain rate and overall dislocation density, as shown in Equation (4):

(4) $$\rho_{m}^{\alpha }=c_{3}{\bar{\rho }}^{\alpha }{}^{c_{4}}$$

where $c_{3}$ and $c_{4}$ are fitting constants $c_{4}=A_{1}/lnZ+B_{1}$ and Z. is Zener–Holloman parameter $Z=\dot{\varepsilon }\cdot exp\left(Q_{Act}/R/T\right)$.

#### 2.2.2. Kinetic Equation of Grain Boundary Sliding

In order to visually analyse the GBS mechanism during hot deformation, this paper adopted the Gifkins model [24] and divided each grain into two parts: the grain core and the GB region. The actual GB thickness of metallic materials is approximately 0.5 nm [25], and the GB region in the Gifkins model not only includes GBs, but also includes a layer near the GBs that is compatible with GBS. The thickness of the GB region is much larger than the actual thickness. In the finite element simulation that explicitly represents the GB region, the thickness of the GB region is usually between 0.5–1 μm [12,26]. These simulation results provide a reference range for the choice of grain boundary thickness, and the grain boundary thickness is finally determined by the fitting of experimental results such as tensile curve and microstructure evolution.

**Figure 3:**
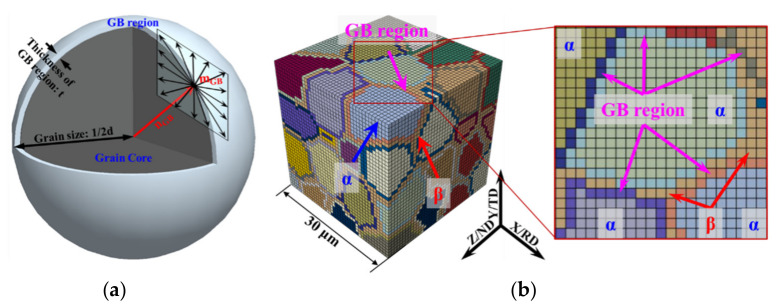
Schematic diagram of motion systems of GB dislocations and the polycrystalline model with the GB region of TA15 samples. (**a**) Motion systems of GB dislocations; (**b**) polycrystalline model with the GB region and the partial enlarged map.

With the increase in deformation temperature and the decrease in strain rate, the vacancy diffusion and dislocation motion occur in the GB regions, leading to the deformation mechanism of GBS [27]. For simplification, this paper assumed that GBS is the result of the coordinated motion of many GB dislocations. The motion system of GB dislocations is shown in Figure 3a, in which the normal direction of the motion plane is the direction vector from the central point of the grain to the points in the GB region.

On the basis of the two-phase polycrystalline model in Figure 2b, the equiaxed α grains were divided into two parts: the grain core and the GB region. The thickness of the GB region was a layer of element, and the element size was 0.75 μm, as shown in Figure 3b. The α phase and β phase in the model only considered the deformation mechanism of the dislocation motion, while the GB region only considered the deformation mechanism of GBS. The GB region in this paper was actually a visualised representation of GBS, therefore, this paper does not further distinguish between the GBs and phase boundaries.

Assuming GBS as the result of the coordinated motion of GB dislocations, it can also be described by a kinetic equation similar to Equation (1). The difference is that the effect of grain size or GB curvature needs to be considered in the kinetic equation of GBS, as shown in Equation (5):

(5) $${\dot{\gamma }}_{GBS}^{\alpha }={\left[\frac{c_{1GBS}\rho_{mGBS}^{\alpha }}{d^{1/2}}exp\left(-\frac{Q_{Act}}{RT}\right)\left|\frac{\tau_{GBS}^{\alpha }}{\tau_{GBSpass}^{\alpha }}\right|\right]}^{1/m}sign\left(\tau^{\alpha }\right)$$

where $c_{1GBS}$ is a fitting constant, $\rho_{mGBS}^{\alpha }$ is the mobile GB dislocation density, $\rho_{mGBS}^{\alpha }=c_{3}{\bar{\rho }}_{GB}^{\alpha }{}^{c_{4}}=c_{3}{\left(\rho_{GB}^{\alpha }/\rho_{0}^{\alpha }\right)}^{c_{4}}$, d is the equivalent diameter of grain and $\tau_{GBSpass}^{\alpha }$ is the motion resistance of GB dislocations $\tau_{GBSpass}^{\alpha }=c_{2GBS}{\bar{\rho }}_{GB}{}^{0.5}=c_{2GBS}{\left(\rho_{GB}/\rho_{0}\right)}^{0.5}$.

#### 2.2.3. Model of Dynamic Recrystallisation

The Kocks–Mecking equation is usually used to predict the evolution of dislocation density during the hot deformation of metals, which includes the deformation-induced dislocation multiplication and DRV-induced dislocation annihilation [12]. Furthermore, the influence of static recovery (SRV) on the evolution of dislocation density must be considered, as shown in Equation (6):

(6) $${\dot{\rho }}^{\alpha }=c_{5}\sqrt{\rho^{\alpha }}{\dot{\gamma }}^{\alpha }-c_{6}\rho^{\alpha }{\dot{\gamma }}^{\alpha }-c_{7}\rho^{\alpha }$$

where $c_{5}$ is a fitting constant related to the dislocation multiplication, $c_{6}$ is a fitting constant related to the DRV-induced dislocation annihilation and $c_{7}$ is a fitting constant related to the SRV-induced dislocation annihilation.

$c_{6}$ represents the rate of DRV-induced dislocation annihilation, and its relationship with the deformation temperature, strain rate and deformation activation energy is shown in Equation (7) [12]. In the same way, the expression of $c_{7}$ is shown in Equation (8):

(7) $$c_{6}=c_{60}\cdot Z^{-1/L}$$

(8) $$c_{7}=c_{70}\cdot Z^{-1/L}$$

where $c_{60}$, $c_{70}$ and L are the fitting constants.

When the accumulated dislocation density near the initial GBs reaches the critical dislocation density, the local GB bows out and forms a recrystallised nucleation. The critical dislocation density $\rho_{c}$ is shown in Equation (9) [12]:

(9) $$\rho_{c}={\left[\frac{20Q_{GB}\dot{\varepsilon }}{3blMQ_{Dis}}\right]}^{1/3}$$

where $Q_{GB}$ is the GB energy per unit area, $\dot{\varepsilon }$ is the macroscopic strain rate, b is the Burgers vector, l is the dislocation mean free path, $l=1000/\sqrt{\rho }$, M is the GB mobility, $M=M_{0}exp\left(-\frac{Q_{Act}}{RT}\right)$, $Q_{Dis}$ is the dislocation line energy and $Q_{Dis}=\mu b^{2}/2$, μ is the shear modulus.

The statistical nucleation rate is adopted to control the number of nuclei, as shown in Equation (10) [12]:

(10) $$\dot{n}=c_{8}{\dot{\varepsilon }}^{m}exp\left(-\frac{Q_{Act}}{RT}\right)$$

where $c_{8}$ is a fitting constant.

The essence of the recrystallised nucleation growth is the GB migration driven by the deformation energy storage. The GB migration speed depends on the GB mobility and the net driving force, as shown in Equation (11):

(11) V=MP

where P is the net driving force for GB migration.

The GB migration consumes the deformation energy storage, while increasing GB energy. Therefore, the net driving force for GB migration is shown in Equation (12):

(12) $$P=1/2\mu b^{2}\Delta \rho -\frac{2\gamma_{s}}{r}$$

where Δρ is the dislocation density difference between the matrix grain and the recrystallised grain, $\gamma_{s}$ is the GB energy of the recrystallised grain and r is the equivalent radius of the recrystallised grain.

GB energy has a linear relationship with the misorientation angle, as shown in Equation (13):

(13) $$\gamma_{s}=\frac{\mu b\theta_{GB}}{4\pi \left(1-\nu \right)}$$

where ν is the Poisson ratio and $\theta_{GB}$ is the misorientation angle of the GB.

**Figure 4:**
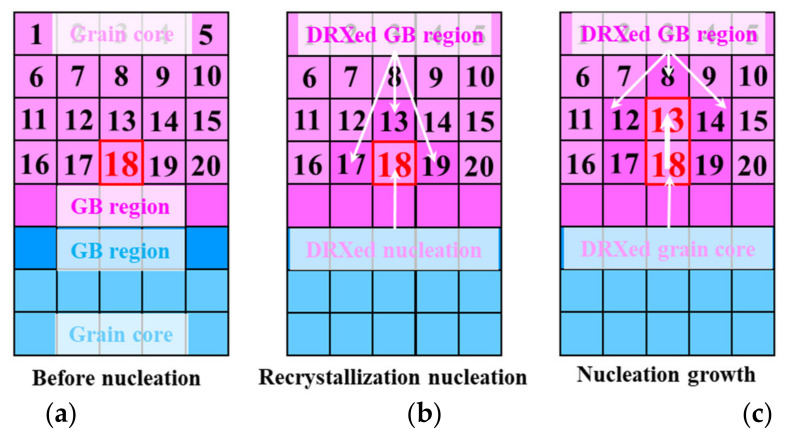
Switch law of recrystallised elements in CPFEM. (**a**) Before nucleation; (**b**) recrystallisation nucleation; (**c**) nucleation growth.

Based on the CA model, the switch law of recrystallised elements in the crystal plasticity finite element model (CPFEM) is shown in Figure 4. When the accumulated dislocation density of an element near the GBs reaches the critical dislocation density, the element, for example element 18, becomes a recrystallised nucleation, and the corresponding adjacent elements (element 13, 17, 19) are transformed into a GB region, as shown in Figure 4a,b. The driving force of the recrystallised nucleation growth basically depends on the dislocation density difference between the recrystallised element (element 18) and the adjacent matrix elements (element 8, 12, 14, 16, 20). The GB migration distances in all directions S are calculated by Equation (14). When S is greater than the distance between the recrystallised element and the corresponding matrix element, the recrystallised nucleation grows up in this direction. As the DRX nucleation grows, the corresponding switch law of nucleation growth is shown in Figure 4c.

(14) $$S=\underset{t}{\sum }V\Delta t$$

where Δt is the time increment.

The dislocation density of the recrystallised grains were assigned the initial dislocation density of the sheet used in this paper, because the sheet was almost entirely composed of recrystallised grains. The orientations of the recrystallised grains were not artificially changed. The recrystallised grains accumulated a large amount of dislocation density caused by the dislocation motion. Due to the grain rotation caused by the dislocation motion, there misorientation occurred between the recrystallised grains and the matrix grains.

Because of the low fraction and the lamellar distribution of the β phase of the TA15 titanium alloy, this paper only considered the DRX of the α phase.

#### 2.2.4. Model of Void Evolution

The ductile fracture of metallic materials usually includes the three stages of void nucleation, growth and coalescence. During the polycrystalline deformation process, the local stress concentration will lead to void nucleation [17], and the higher slip activity results in the large plastic deformation in the matrix material around the void causing higher void growth [15]. Therefore, this paper referred to the cumulative plastic strain energy model [28], and established a void nucleation model, considering the influence of stress and strain as shown in Equation (15). When the superposition of the product of the equivalent stress and the shear strain of each slip system exceeds a certain critical value, void nucleation occurs. Moreover, the relationship between the critical value of void nucleation and the deformation temperature and strain rate was established.

(15) $$\overset{N}{\underset{\alpha =1}{\sum }}\int_{0}^{\gamma }T_{eff}^{\alpha }d\gamma^{\alpha }=C_{Void}=f\left(\dot{\varepsilon },T\right)$$

where $T_{eff}^{\alpha }$ is the equivalent stress of the slip system α and $C_{Void}$ is the critical value of void nucleation $C_{Void}=f\left(\dot{\varepsilon },T\right)=A_{2}lnZ+B_{2}$.

The equivalent stress should not only consider the stress value but also the stress state. The tensile normal stress can simply be incorporated while the compressive normal stress is not considered. At the same time, the influence of shear stress on the void nucleation is considered through a scale factor of less than 1. The equivalent stress of the slip system α is shown in Equation (16) [29]:

(16) $$T_{eff}=\sqrt{T_{n}{}^{2}+\delta T_{t}{}^{2}}$$

where $T_{n}$ is the normal stress of the slip system α, $T_{t}$ is the shear stress of the slip system α and δ is a scale factor δ=0.7 [30].

The strength of the elements defined as voids were artificially assigned a low value (5 MPa), which hardly contributed to the overall strength of the specimen.

### 2.3. Parameter Determination

The parameters of the crystal plasticity constitutive model were crucial to the simulated results, which were mainly obtained by referring to the literature or by fitting to experimentally determined hot tensile stress–strain data. All parameters are summarized in Table 1.

The shear modulus of the α phase and β phase of titanium alloys decreased with the increasing temperature, as shown in Equation (17) [31]:

(17) $$\mu_{\alpha ,\beta }=\mu_{0\alpha ,\beta }\left[1+\left(\frac{T-300}{T_{M}}\right)\left(\frac{T_{M}}{\mu_{0\alpha ,\beta }}\frac{d\mu_{\alpha ,\beta }}{dT}\right)\right]$$

where $\mu_{0\alpha ,\beta }$ is the shear modulus of α or β phase of titanium alloys at room temperature (GPa)*,* $\mu_{0\alpha }=43.6$, $\mu_{0\beta }=20.5$, $T_{M}$ is the melting point of the titanium alloys, $T_{M}=1998\;K$ and $\frac{T_{M}}{\mu_{0\alpha ,\beta }}\frac{d\mu_{\alpha ,\beta }}{dT}$ is the coefficient representing the effect of temperature on the shear modulus, $\frac{T_{M}}{\mu_{0\alpha }}\frac{d\mu_{\alpha }}{dT}=-1.2$, $\frac{T_{M}}{\mu_{0\beta }}\frac{d\mu_{\beta }}{dT}=-0.5$.

The elastic modulus matrices of the α and β phases of the titanium alloy also decreased with the increasing temperature, which were approximatively obtained by Equation (18) [32]:

(18) $$C_{ij}=C_{ij0}\frac{\mu \left(T\right)}{\mu_{RT}}$$

where $C_{ij0}$ is the parameter in the elastic modulus matrices of the α and β phases of titanium alloy at room temperature (GPa)*,* $C_{110}^{\alpha }=141$, $C_{120}^{\alpha }=76.9$, $C_{130}^{\alpha }=57.9$, $C_{330}^{\alpha }=163$, $C_{440}^{\alpha }=48.7$, $C_{110}^{\beta }=135$, $C_{120}^{\beta }=113$, $C_{440}^{\beta }=54.9$, $\mu \left(T\right)$ is the shear modulus of titanium alloys at the temperature of T and $\mu_{RT}$ is the shear modulus of titanium alloys at room temperature.

The dislocations of metal materials are generally divided into two categories: geometrically necessary dislocations (GNDs) and statistically stored dislocations (SSDs). GNDs, mostly, are the dislocations with the same signs and arranged regularly, and SSDs, mostly, are the dislocations with the opposite signs and randomly distributed [33]. The TA15 titanium alloy sheet used in this paper underwent a recrystallised annealing process, and the dislocations with the opposite signs were eliminated through SRV. In addition, the GND density dominated the total dislocation density in the small and medium plastic deformation of the polycrystalline metals and alloys [33,34]. The TA15 sheet used in this paper contained hardly any deformed structures. Therefore, it was reasonable to assume that the remaining dislocations of the TA15 titanium alloy sheet were mainly GNDs. The GNDs density $\rho_{GND}$ is usually approximately linear with the average misorientation θ, as shown in Equation (20), which can be directly replaced by the Kernel average misorientation (KAM) in the EBSD data [34]. The average KAM inside the α grains of the TA15 titanium alloy sheet was 0.27°. The calculated initial dislocation density was 6 × 10^14^ m^−2^, which is approximately consistent with the results in the literature [35]. In addition, the dislocation density in the GB region also showed a linear relationship with the misorientation angle, which can also be calculated by Equation (19).

(19) $$\rho_{GND}=2\theta /u/b$$

where θ is the average misorientation and u is the scan size of EBSD.

During the hot deformation process of the α phase of the titanium alloys, the CRSSs ratio of slip systems was approximately 1:0.7:3 (basal 〈a〉: prismatic 〈a〉: pyramidal 〈c+a〉). The CRSSs of the β phase during the hot deformation process were similar. In a large deformation temperature range, the deformation resistance ratio of the β phase to α phase was approximately 1/2–1/5 [10]. The difference in slip resistance between the slip systems of the α and β phase was represented by the coefficient $c_{2}$, and the ratio between the slip systems was finally determined by the tensile curve fitting.

## 3. Results

### 3.1. Model Verification

**Figure 5:**
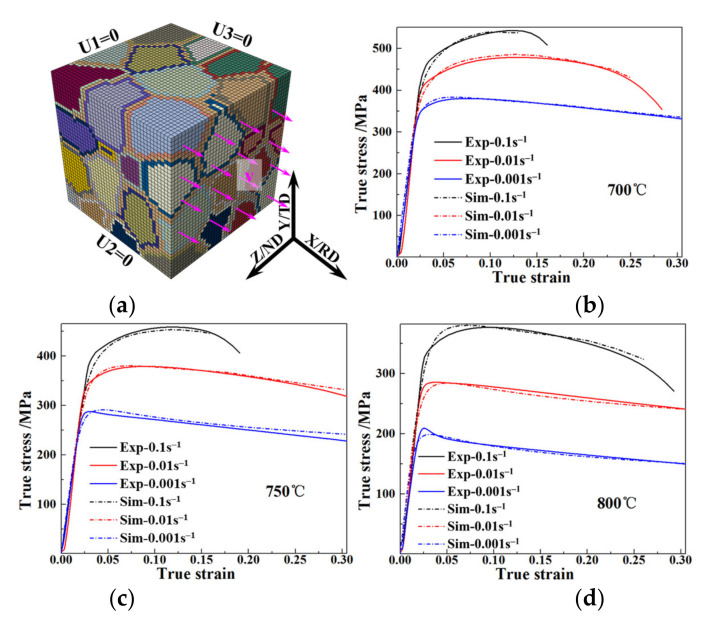
Boundary conditions of polycrystalline model and the comparison of simulated and experimental true stress–strain of TA15 sheet during hot tension. (**a**) Boundary condition of polycrystalline model; true stress–strain at 700 °C (**b**), 750 °C (**c**) and 800 °C (**d**).

The interrupted tensile tests of the TA15 sheet along the RD were performed on an INSTRON 5500R test machine from 700–800 °C with the initial strain rate from 0.1–0.001 s^−1^. The true stress–strain curves are shown in Figure 5. Under the deformation conditions with low temperature and high strain rate, the TA15 sheet exhibited deformation hardening followed by necking and fracture. Under the deformation conditions of high temperature and low strain rate, the TA15 sheet exhibited the dynamic softening induced by DRV and DRX. At such non-superplastic forming conditions, strain and strain rate dual hardening [36] and softening caused by necking and microstructure evolution coexisted.

Figure 5a shows the boundary conditions of the polycrystalline finite element model. With the increase in deformation temperature from 700 °C to 800 °C, the volume fraction of the β phase increased from 11% to 14%, and the slight increase was not considered in CPFEM. The X–Y, Y–Z and X–Z planes of the polycrystalline model were set to U3 = 0, U1 = 0 and U2 = 0, respectively. The tensile deformation was implemented by setting a constant velocity on the front plane along the X-axis (RD). The comparison of simulated and experimental results of the true stress–strain is shown in Figure 5b–d, in which the close agreement proves the effectiveness of the constitutive model, model parameters and polycrystalline model established in this paper.

### 3.2. Microscopic Heterogeneous Deformation

**Figure 6:**
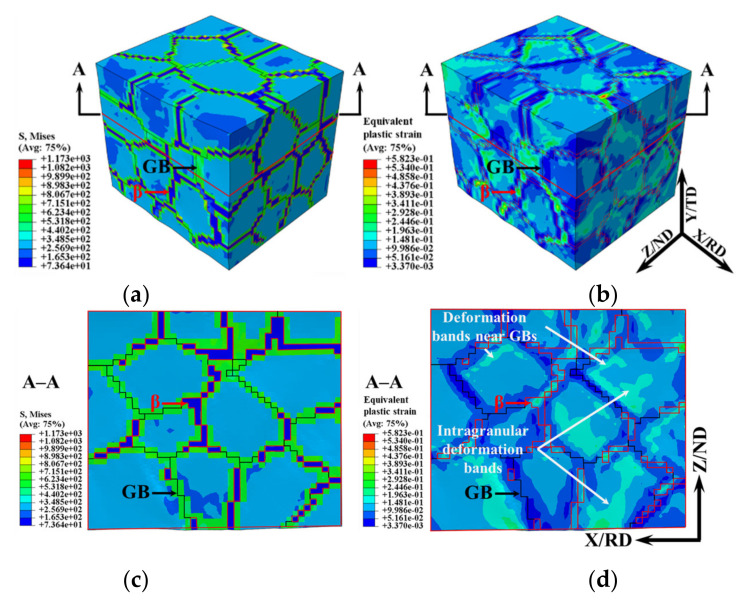
Simulated results of stress and strain of TA15 sample during hot tension (750 °C–0.01 s^−1^–true strain 0.1). 3D stress (**a**) and strain (**b**); 2D stress (**c**) and strain (**d**) in cross section A–A.

Under the hot tensile deformation conditions of 750 °C–0.01 s^−1^–true strain 0.1, the simulated microscopic stress and strain distribution of the TA15 sample are shown in Figure 6, in which the area surrounded by the red lines is the β phase, and the black lines are the GBs. The strain is distributed in a sequential manner, while the stress is distributed in a mutational manner.

The strength difference between the constituent structures (α phase, β phase and GB regions) led to the uneven distribution of the microscopic stress and strain. According to the constitutive model parameters shown in Table 1, under the hot deformation conditions of the TA15 titanium alloy selected in Section 3.1, the strength of the α phase was nearly twice that of β phase (${\bar{\sigma }}_{\alpha }=2{\bar{\sigma }}_{\beta }$). Due to the relatively low forming temperature, high strain rate and large grain size, the strength of the GB regions was always greater than that of the α phase (${\bar{\sigma }}_{GB}>{\bar{\sigma }}_{\alpha }$).

The strength difference between the various regions of the TA15 polycrystalline model (${\bar{\sigma }}_{GB}>{\bar{\sigma }}_{\alpha }>{\bar{\sigma }}_{\beta }$) resulted in the cloud image of the microscopic stress distribution being clearly divided into three regions: low-stress β phase, high-stress GB region and the intermediate-stress α phase as shown in Figure 6a,c. Generally speaking, due to the difference in grain orientation, there were also differences in the distribution of microscopic stress and strain between the α phase grains. However, due to the greater difference in the strength between the three structures, the difference between the α grains was not as clear.

Deformation bands are clearly appeared on the cloud map of the microscopic strain distribution, and the deformation bands were at nearly 45° to the tensile direction, as shown in Figure 6d. The β phase had the lowest strength and deformed preferentially, especially the β phase which was approximately 45° to the tensile direction. In order to adapt to the deformation of the β phase, the deformation band was approximately parallel to the deformed β phase which also appeared near the GBs in the α grains. In addition, a deformation band formed at the triple junction to accommodate deformation inhomogeneity caused by the β phase. Due to the small volume fraction of the β phase and the high strength of the GB region, some deformation bands did not originate from the triple junctions, but directly penetrated the α grains. Therefore, there were three high strain regions: the β phase, the intragranular deformation band and the deformation band near the GBs.

**Figure 7:**
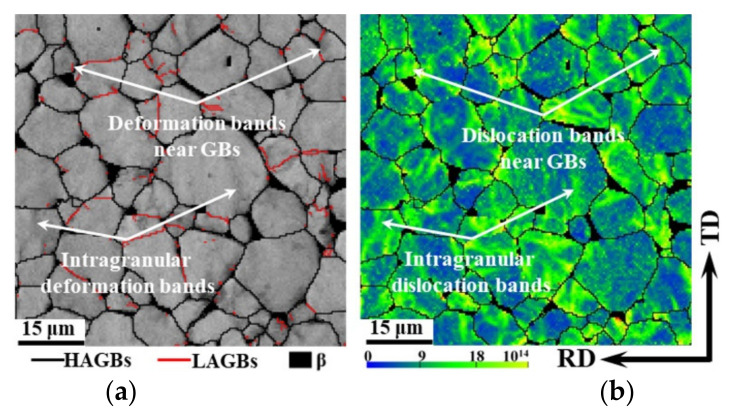
Image quality (IQ) map and GNDs density map of the TA15 tensile samples (750 °C–0.01 s^−1^–true strain 0.1). (**a**) IQ; (**b**) GNDs density.

Figure 7a and Figure 7b, respectively, show the distribution of the image quality (IQ) and GNDs density of the TA15 tensile samples (750 °C–0.01 s^−1^–true strain 0.1), in which the black area represents the β phase, the black line represents the HAGBs, and the red line represents the LAGBs. The IQ map qualitatively describes the distribution of the deformation bands, the lower the IQ value, the more severe the deformation [37]. Similar to the simulated results of the microscopic strain shown in Figure 6d, with the exception of the β phase, there were two low IQ regions: the intragranular regions and the regions near the GBs. The GNDs density was consistent with the microscopic strain distribution of the TA15 tensile sample, and the dislocation density near the β phase was usually high. The close agreement of the simulated and experimental results proves the effectiveness of the crystal plasticity model.

**Figure 8:**
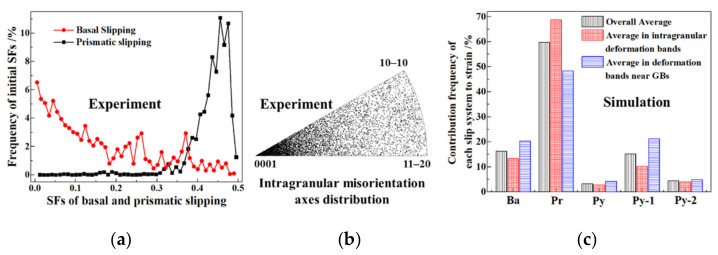
Analysis of activated slip systems of TA15 sample during hot tension (750 °C–0.01 s^−1^–true strain 0.1). (**a**) Initial distribution of basal and prismatic SFs; (**b**) intragranular misorientation axes distribution after deformation in experiment; (**c**) contribution frequency of each slip system to strain in simulation.

Among the five slip systems in the α phase of the titanium alloys, the basal and prismatic slip systems are usually regarded as the dominant slip mechanisms due to the relatively low CRSS during hot deformation. The frequencies of the initial basal and prismatic SFs are shown in Figure 8a, in which the frequencies of the low basal SF and high prismatic SF are high due to the strong basal texture shown in Figure 2c. Thus, the prismatic slipping can be regarded as the most pronounced slip mode during the hot tension along the RD. The analysis of the intragranular misorientation axes distribution after deformation also verifies this statement, in which the distribution of the intragranular misorientation axes on the [0001] orientation resulted from the prismatic slipping as shown in Figure 8b.

The discrete distribution of the intragranular misorientation axes, with the exception of the [0001] orientation, indicated the occurrence of multiple slipping. In order to quantitatively analyse the contribution frequency of each slip system to strain, the corresponding shear strains in the overall model, intragranular deformation bands and deformation bands near the GBs were extracted and normalised as shown in Figure 8c. Apart from the predominant prismatic slipping, the basal and pyramidal-1 slipping played a non-negligible role in plastic deformation. The basal and prismatic slipping had the common slip direction [$11\bar{2}$0], which could not induce any elongation along the c-axis. Consequently, in order to accommodate strain in the c-direction, the pyramidal-1 or pyramidal-2 slip systems must be activated.

As for the intragranular deformation band, the contribution of prismatic slipping to the deformation increased, and the effect of basal and pyramidal-1 slipping was suppressed. However, as for the deformation bands near the GBs, an opposite pattern appeared. It can be supposed that, due to the constraint of the GBs on the grain rotation resulting from dislocation slipping, the multiple slipping must be activated to meet the higher requirement of deformation compatibility near the GBs.

### 3.3. Dynamic Recrystallisation

**Figure 9:**
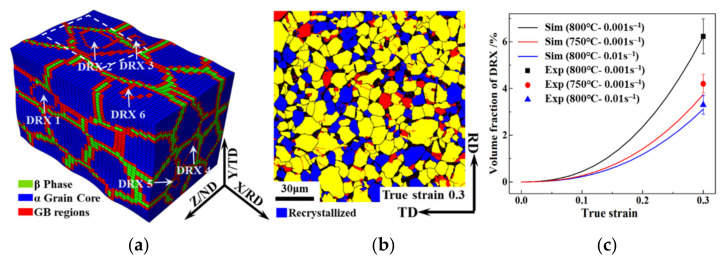
Simulated and experimental results of the recrystallised grains and fractions of the TA15 tensile samples (800 °C–0.001 s^−1^–true strain 0.3). simulated (**a**) and experimental (**b**) recrystallised grains; (**c**) simulated and experimental recrystallised fractions.

The initial microstructure of the TA15 sheet was almost entirely recrystallised. It could not be determined whether the recrystallised grains of the tensile specimen were attributed to the initial recrystallised microstructure or the recrystallised microstructure induced by the tensile deformation. Therefore, the recrystallised grains with a size of more than 5 μm in the tensile sample were defined as the initial recrystallizesed microstructure. Under the tensile deformation condition of 800 °C–0.001 s^−1^–true strain 0.3, the simulated and experimental results of the recrystallised grains and fractions are shown in Figure 9, in which the simulated results agreed well with the experimental data. Due to the low initial deformation energy storage of the undeformed TA15 sheet, the recrystallisation fraction was approximately 6%, as shown in Figure 9c.

**Figure 10:**
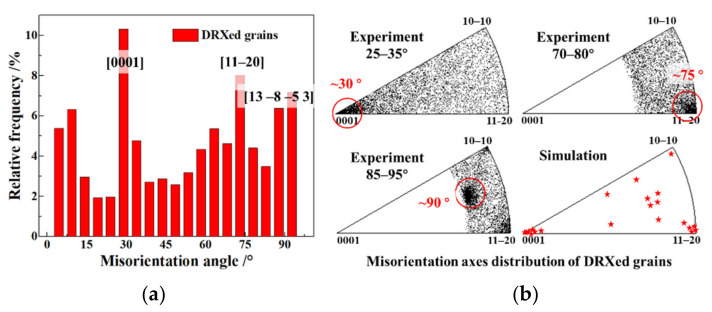
Analysis of GBs misorientation of recrystallised grains of the TA15 tensile samples (800 °C–0.001 s^−1^–true strain 0.3). (**a**) Misorientation; (**b**) experimental and simulated distribution of misorientation axes.

The activated dislocations gradually accumulated near the GBs and formed a recrystallised nucleation. Therefore, the grain rotation resulting from dislocation slipping had a crucial effect on the GBs misorientation and the orientation of the recrystallised grains. The analysis of the GBs misorientation of the recrystallized grains of the TA15 tensile samples (800 °C–0.001 s^−1^–true strain 0.3) are shown in Figure 10. The misorientation distribution manifested in three misorientation peaks at 30°, 75° and 90° as shown in Figure 10a. The experimental and simulated distribution of misorientation axes concentrated on the [0001], [$11\bar{2}$0], and [$13\;\bar{8}\;\bar{5}\;3$] orientations as shown in Figure 10b, in which the consistency further proves the accuracy of the simulation. Based on the above analysis, one can conclude that the GBs of recrystallised grains distributed mainly in three misorientations, namely [0001] 30°, [$11\bar{2}$0] 75° and [$13\;\bar{8}\;\bar{5}\;3$] 90°. The [0001]-axis and [$13\;\bar{8}\;\bar{5}\;3$]-axis misorientations were due to the activation of the prismatic and pyramidal-1 slip systems, respectively. Yet, the [$11\bar{2}$0]-axis misorientation was attributed to the basal or pyramidal-2 slipping [38]. The analysis of the GBs misorientation of recrystallised grains was roughly consistent with the result of the activated slip systems shown in Figure 8c.

**Figure 11:**
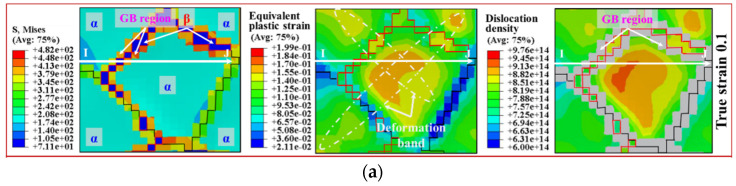

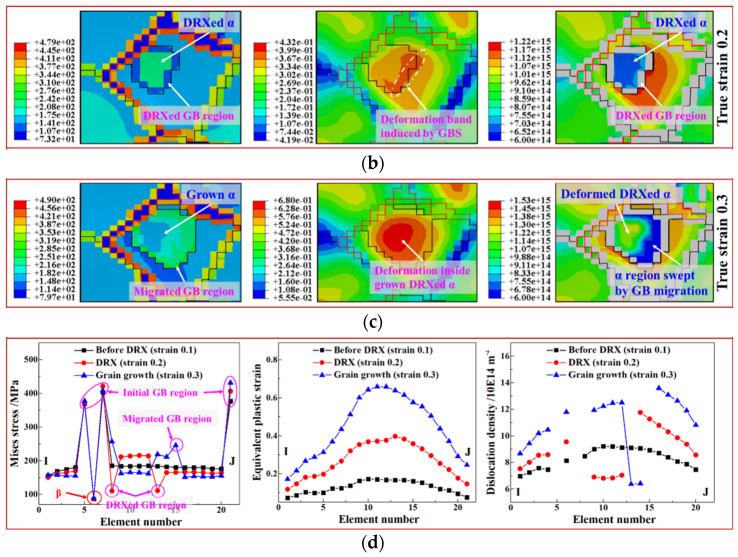
Simulated distribution of stress, strain and dislocation density of DRX in the white dashed box in the Figure 9a (800 °C–0.001 s^−1^). (**a**) True strain of 0.1; (**b**) true strain of 0.2; (**c**) true strain of 0.3; (**d**) line chart of stress, strain and dislocation density under different DRXed stages.

Under the tensile deformation condition of 800 °C–0.001 s^−1^ of the TA15 tensile sample, the simulated distribution of stress, strain and dislocation density of DRX behaviour in the white dashed box of Figure 9a are shown in Figure 11. The dislocation density in the GB region, due to the significantly greater value than that of the α and β phases, is not shown on the distribution cloud map of dislocation density, as shown in the gray area of Figure 11.

When the true strain was 0.1, similar to the simulated results of microscopic strain shown in Figure 6d, there were two intragranular deformation bands, as shown in Figure 11a, and the distribution of dislocation density was consistent with the strain distribution. When the accumulated dislocation density near the GB region exceeded the critical value, the recrystallised nucleation occurred, as shown in Figure 11b. The dislocation density inside the recrystallised grain was usually low, which significantly reduced the mobile dislocation density, so that the stress inside the recrystallised grain was slightly increased. The increased stress limited the deformation inside the recrystallised grain. However, the small grain size of recrystallised grains is prone to GBS, which makes the deformation bands transfer to the GB region of the recrystallised grain and improves the uniformity of microscopic deformation. The fine defect-free recrystallised grain is similar to a hard particle, which is prone to grain rotation through the GBS mechanism and is not prone to intragranular deformation through the dislocation motion mechanism.

When the recrystallised grains grow up, the dislocation density of the region swept by the GB migration is significantly reduced. The increased grain size significantly inhibits the GBS mechanism, so that the deformation is transferred to the inside of the recrystallised grain, as shown in Figure 11c. The strain and dislocation density inside the recrystallised grains is significantly increased.

### 3.4. Void Evolution

**Figure 12:**
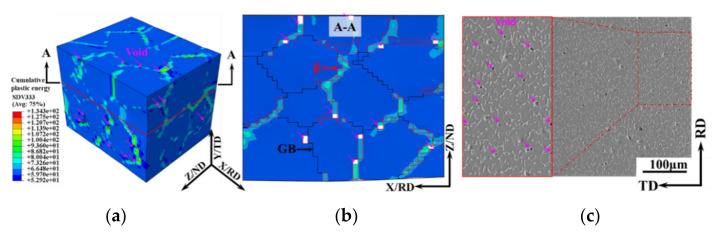
Simulated and experimental results of void distribution of the TA15 sheet during hot tension (750 °C–0.1 s^−1^–true strain 0.1). (**a**) 3D map of simulated void distribution; (**b**) cross-section of A–A; (**c**) SEM map.

Figure 12 shows the simulated and experimental results of the void distribution of the TA15 sheet during hot tension (750 °C–0.1 s^−1^–true strain 0.1). The close agreement between the experimental and simulated results proves the effectiveness of the crystal plasticity model. The cumulative plastic strain energy of the β phase region was significantly greater than the other regions, and the voids nucleated at the phase boundary first.

**Figure 13:**
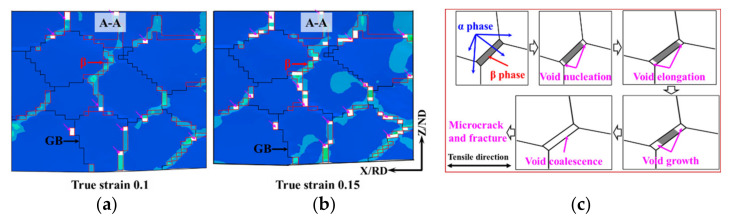
Simulated results and schematic of void evolution during hot tension of the TA15 sample (750 °C–0.1 s^−1^). (**a**) True strain 0.1; (**b**) true strain 0.15; (**c**) schematic of the void evolution.

Figure 13 shows the simulated results and the schematic of the void evolution during hot tension of the TA15 sample (750 °C–0.1 s^−1^). The voids first nucleated at the phase boundary and subsequently elongated along the tensile direction. Simultaneously, when the region around the voids met the void nucleation criterion, the voids grew. Finally, when the grown voids expanded and meet in the β phase region, microcracks that were 45° to the tensile direction were formed.

Generally speaking, the local stress concentration led to void nucleation. However, due to the prominent strength difference and compatibility requirement between the β phase and the GB region, the β phase experienced significantly greater strain as shown in Figure 6. Therefore, in this study, in the simulation of a near-α titanium alloy, the strain distribution played a major role in the void evolution.

### 3.5. Effect of Hot Deformation Conditions on Microscopic Deformation

The *microscopic heterogeneous deformation* of the TA15 titanium alloy during non-superplastic hot deformation was determined by the strength difference between the constituent structures. According to the constitutive model parameters shown in Table 1, under the non-superplastic hot deformation conditions of the TA15 titanium alloy studied in this paper, the strength of the α phase was nearly twice that of the β phase (${\bar{\sigma }}_{\alpha }=2{\bar{\sigma }}_{\beta }$). Therefore, there were five strength relationships between the three structures, as shown in Table 2. ${\bar{\sigma }}_{GB}={\bar{\sigma }}_{\alpha }=2{\bar{\sigma }}_{\beta }$ indicates that the strength of the GB region was equivalent to that of the α phase, that is, the resistance of the dislocation motion of the α phase and the GBS was nearly equivalent. ${\bar{\sigma }}_{GB}=0.5{\bar{\sigma }}_{\alpha }={\bar{\sigma }}_{\beta }$ indicates that the strength of the GB region was nearly equivalent to that of the β phase.

According to the kinetic model of dislocation motion and GBS, the simplified strength expressions of the GB region and the α phase of the TA15 samples could be obtained as shown in Equations (20) and (21). The strength ratio of the GB region and the α phase is shown in Equation (22). Due to the low initial dislocation density of the TA15 sheet, the relative dislocation density of the α phase was set to 1 (${\bar{\rho }}_{\alpha }=1$).

(20) $${\bar{\sigma }}_{GB}=M{\bar{\tau }}_{GBS}=M\frac{c_{2}}{c_{1GBS}c_{3}}exp\left(\frac{Q_{Act}}{RT}\right){\dot{\gamma }}_{GBS}^{\alpha }{}^{m}d^{1/2}{\bar{\rho }}_{GB}^{0.5-c_{4}}$$

(21) $${\bar{\sigma }}_{\alpha }=M{\bar{\tau }}_{\alpha }=M\frac{c_{2}}{c_{1}c_{3}}exp\left(\frac{Q_{Act}}{RT}\right){\dot{\gamma }}^{\alpha }{}^{m}{\bar{\rho }}_{\alpha }^{0.5-c_{4}}$$

(22) $${\bar{\sigma }}_{GB}/{\bar{\sigma }}_{\alpha }=\frac{c_{1}}{c_{1GBS}}{\bar{\rho }}_{GB}^{0.5-c_{4}}d^{1/2}$$

where M is the Taylor factor.

**Figure 14:**
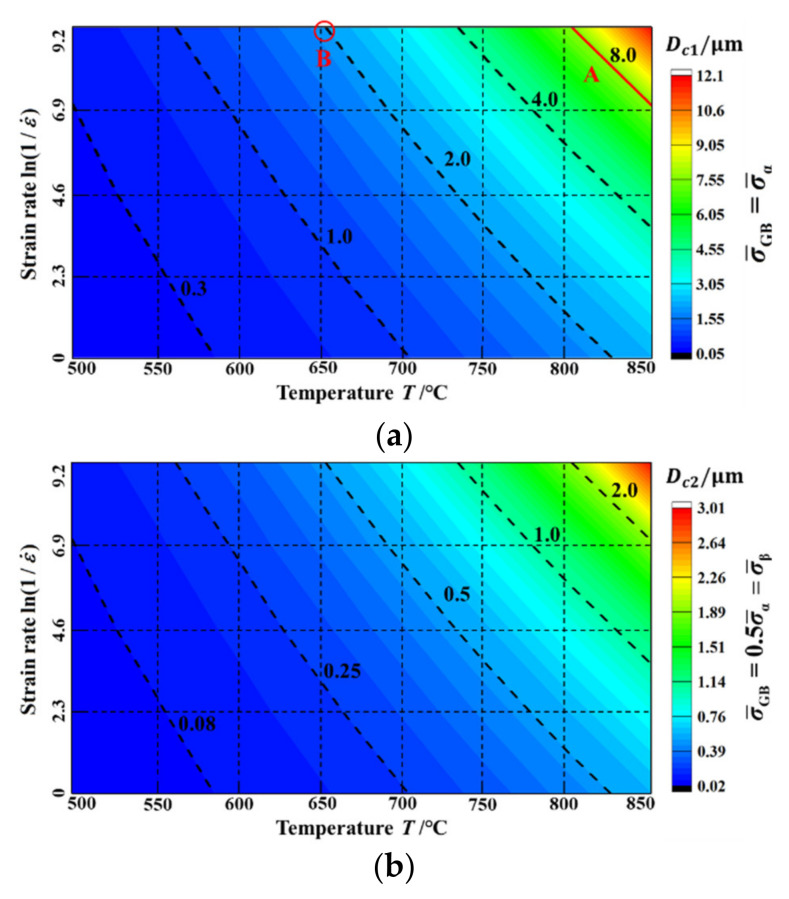
Critical grain size of TA15 samples when the strength of the GB region is equal to the α phase ($D_{c1}$) or the β phase ($D_{c2}$) under different hot deformation conditions. (**a**) ${\bar{\sigma }}_{GB}={\bar{\sigma }}_{\alpha }=2{\bar{\sigma }}_{\beta }$, (**b**) ${\bar{\sigma }}_{GB}=0.5{\bar{\sigma }}_{\alpha }={\bar{\sigma }}_{\beta }$.

According to Equation (22) and the model parameters in Table 1, the critical grain size of the TA15 samples, when the strength of the GB regions were equal to the α phase ($D_{c1}$) or β phase ($D_{c2}$) under the different hot deformation conditions, were calculated according to Equation (23), and the calculated results are shown in Figure 14.

(23) $$D_{c1}={\left(\frac{c_{1GBS}}{c_{1}}{\bar{\rho }}_{GB}^{c_{4}-0.5}\right)}^{2}={\left(0.11\times {\bar{\rho }}_{GB}^{39.5/lnZ-0.15}\right)}^{2}=4D_{c2}$$

When parameter Z decreased the critical grain size $D_{c1}$/$D_{c2}$ gradually increased. For example, when the average grain size of the TA15 samples was 8 μm, only the deformation conditions with parameter Z below line A could realise a GB strength equal to or less than the α phase, as shown in Figure 14a. In addition, during the deformation condition of 650 °C–0.0001 s^−1^, only when the average grain size was less than 2 μm could the strength of the GB region be equal to or less than the α phase, as shown by point B in Figure 14a.

**Figure 15:**
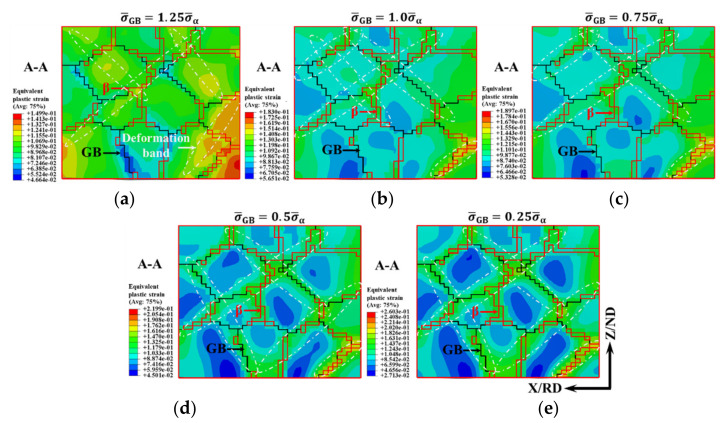
Simulated strain distribution of TA15 tensile samples with different strength relationship of three structures with true strain of 0.1. (**a**) ${\bar{\sigma }}_{GB}=1.25{\bar{\sigma }}_{\alpha }$; (**b**) ${\bar{\sigma }}_{GB}=1.0{\bar{\sigma }}_{\alpha }$; (**c**) ${\bar{\sigma }}_{GB}=0.75{\bar{\sigma }}_{\alpha }$; (**d**) ${\bar{\sigma }}_{GB}=0.5{\bar{\sigma }}_{\alpha }$; (**e**) ${\bar{\sigma }}_{GB}=0.25{\bar{\sigma }}_{\alpha }$.

The simulated results of the microscopic strain distribution of the TA15 tensile samples with a true strain of 0.1 with a different strength relationship are shown in Figure 15, in which the area surrounded by the red line is the β phase, the black line is the GBs, and the section A–A is shown in Figure 6. Due to the relatively low deformation temperature considered in this paper, the slight increase of the volume fraction of the β phase was not considered in CPFEM.

When ${\bar{\sigma }}_{GB}=1.25{\bar{\sigma }}_{\alpha }=2.5{\bar{\sigma }}_{\beta }$, due to the same strength relationship of the three structures as Section 3.2, the distribution of microscopic strain was similar, as shown in Figure 15a and Figure 6d. There were three high strain regions: the β phase region, the intragranular deformation bands, and the deformation band near the GBs. When ${\bar{\sigma }}_{GB}=1.0{\bar{\sigma }}_{\alpha }$ or ${\bar{\sigma }}_{GB}=0.75{\bar{\sigma }}_{\alpha }$, the deformation bands were mainly distributed in the β phase and GB regions near the β phase. Compared with Figure 15a, the intragranular strain was significantly reduced. When ${\bar{\sigma }}_{GB}=0.5{\bar{\sigma }}_{\alpha }$ or ${\bar{\sigma }}_{GB}=0.25{\bar{\sigma }}_{\alpha }$, the deformation bands were mainly distributed in the GB regions and β phase, and almost no deformation occurred in the central region of the α grains.

## 4. Discussion

### 4.1. Transformative Accommodation Mechanisms of GBS

**Figure 16:**
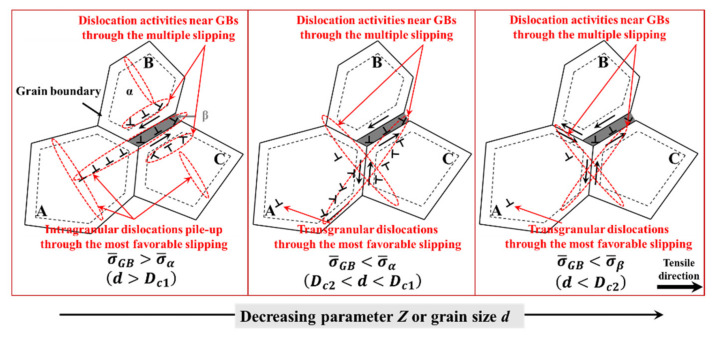
Schematic of transformative accommodation mechanisms of dislocation motion associated with GBS under different hot deformation conditions of near-α titanium alloys.

Atomic diffusion and dislocation motion are regarded as the main accommodation mechanisms of GBS during hot metal forming. Hot in-situ tensile and shear deformation tests in the temperature range from 700–900 °C were performed to study the mechanisms responsible for the superplastic effect in Ti-6Al-4V with the similar equiaxed microstructure to that of Figure 2a. The surface observations proved that dislocation motion (either α or β) is the main phenomenon accountable for the accommodation process of GBS [31]. Therefore, according to the previous simulated and experimental results, this section discusses the transformative accommodation mechanisms of dislocation motion associated with GBS under different hot deformation conditions, as shown in Figure 16.

With the decrease in parameter *Z* or grain size, the plastic strain contribution from GBS gradually increased. As for the two-phase titanium alloys, regardless of hot deformation condition, the weaker β phase made a greater contribution to the accommodation of the preferential GBS compared to the stronger α phase and, thus, dislocation activities in the β phase occurred from the early stage of deformation. The higher the volume fraction of the β phase, the more crucial the role on the accommodation mechanisms of GBS [31]. The particular emphasis of this paper is placed on the hot forming of titanium alloys with relatively low temperature, where the volume fraction of the β phase increases insignificantly. Therefore, the effect of the volume fraction of the β phase on the accommodation mechanisms of GBS was not discussed in detail.

The accommodation mechanisms of dislocation motion associated with GBS fall into two categories: the intragranular dislocation activities and that near the GBs. GBS causes stress concentrations at triple junctions, which are accommodated by intragranular dislocation activities. When ${\bar{\sigma }}_{GB}$ is greater than ${\bar{\sigma }}_{\alpha }$ ($d>D_{c1}$), the activated dislocations pile up inside the grain. In turn, the dislocations travel across the grain interior. Langdon proposed the equilibrium cell size (*λ*) and discussed the similar accommodation mechanisms of dislocation motion associated with GBS [27]. Alabort et al. calculated the equilibrium cell size (*λ*) of Ti-6Al-4V with the similar equiaxed microstructure to that of Figure 2a, in which the calculated results are in the same order of magnitude as that of Figure 14a [31]. The accumulated dislocations are mainly composed of the most easily activated slip systems, namely the prismatic slipping in this paper, as shown in Figure 8c. Masuda et al. also proposed a similar mechanism, discovering that a transgranular slipping along $\left[1\bar{1}1\right]\left(10\bar{1}\right)$ activated to accommodate the GBS, as the primary slip system in BCC-structured steel [39].

Due to the requirement of microscopic deformation compatibility, GBS should also be accommodated by dislocation activities near the GBs through multiple slipping, as shown in Figure 8c. Many observations suggest that dislocation activities near the GBs are responsible for the accommodation of GBS [40,41,42]. The regions of accommodating dislocations near the GBs are not uniformly thick, which are significantly dependent on the GB geometry and the applied stress conditions. When ${\bar{\sigma }}_{GB}$ was less than ${\bar{\sigma }}_{\alpha }$ ($d<D_{c1}$), the average thickness of the accommodating regions near the GBs decreased with the decreasing parameter *Z* or grain size, as shown in Figure 15. By this time, one may speculate that GB diffusion and grain rotation may have a nonnegligible effect on the accommodation mechanism of GBS.

### 4.2. Transformative Mechanisms of Microstructure Evolution

**Figure 17:**
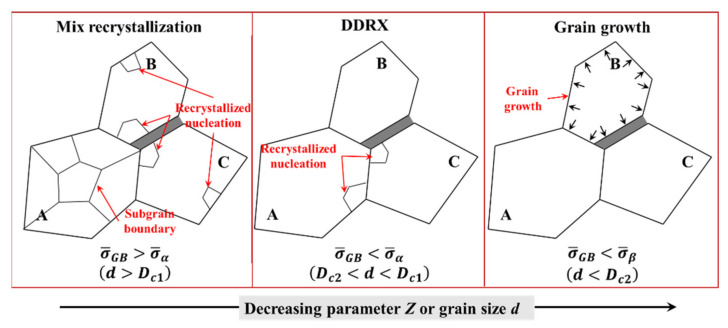
Schematic of transformative mechanisms of microstructure evolution under different hot deformation conditions of near-α titanium alloys.

The microscopic heterogeneous deformation determines the microstructure evolution, and the corresponding evolution under different hot deformation conditions are shown in Figure 17.

When ${\bar{\sigma }}_{GB}$ was greater than ${\bar{\sigma }}_{\alpha }$, there were three high strain regions: the β phase region, the intragranular deformation band and the deformation band near the GBs, as shown in Figure 6 and Figure 13a. The accumulated dislocations in the intragranular deformation bands gradually rearranged to form subgrain boundaries driven by SRV/DRV [37]. Several subgrain boundaries that extended into grain A also formed, thereby dividing grain A into multiple subgrains. The intragranular deformation bands served as the main mechanism for grain fragmentation, i.e., continuous dynamic recrystallisation (CDRX) [39,43]. At the same time, the deformation band near the GBs brought about the dislocation accumulation, which resulted in the GB bowing out to form the nucleation of discontinuous dynamic recrystallisation (DDRX) [44]. Therefore, mixed recrystallisation of CDRX and DDRX appeared.

With the decrease in parameter Z or grain size, when ${\bar{\sigma }}_{GB}$ was less than ${\bar{\sigma }}_{\alpha }$, there were three high strain regions: the β phase region, the GB regions and the α phase adjacent to the GBs, as shown in Figure 15c. In order to adapt to the stimulative GBS mechanism, dislocations began and accumulated in the α phase adjacent to the GBs, which easily induced the nucleation of DDRX.

When ${\bar{\sigma }}_{GB}$ was less than ${\bar{\sigma }}_{\beta }$, the deformation bands were mainly distributed in the GB regions and β phase, as shown in Figure 13e. The accommodating dislocation activities of GBS were suppressed, and almost no dislocation accumulation was formed. The contribution of GBS to the total strain was generally from 50–70%, and the deformation was in the region of superplastic flow [45]. By this time, the main microstructure evolution was grain growth.

### 4.3. Methods to Improve the Macroscopic Plastic Formability

In order to improve the macroscopic plastic formability and microscopic deformation compatibility of difficult-to-form titanium alloys during non-superplastic hot deformation, research can be carried out with regards to two main aspects: the control methods of the initial microstructure and progress of the hot forming process.

It is generally believed that superplastic forming requires high forming temperature (above 0.5 $T_{m}$) and fine phase/grain size (smaller than 10 μm) [27]. According to the calculated results of the selected hot deformation conditions in Figure 14b, when the deformation temperature was relatively low, as long as the grain size was small enough, the superplastic flow was also satisfied. For example, the deformation behaviour of the ultrafine Ti-6Al-4V with an average size of 0.1–0.4 μm at low deformation temperatures (~550 C) was superplastic with a total elongation up to 1000% [46], in which the grain size was in the same order of magnitude as the result in Figure 14b.

In addition, the increase in the β phase volume fraction improved the microscopic deformation compatibility of the titanium alloys during hot deformation. The isothermal hydrogenation treatment significantly reduced the phase transformation temperature of the titanium alloy, so that at a relatively low deformation temperature, the β phase content of the titanium alloy was significantly increased, which improved the macroscopic plastic formability [47].

The DRX behaviour of titanium alloys during hot deformation could relieve the local stress concentration and significantly promote GBS mechanism, which also improved the macroscopic plastic formability [48]. The substantial prior deformed structures of titanium alloys, viz., subgrain boundaries and dislocations, can expedite the occurrence of DRX during hot deformation [38]. The severe plastic deformation and the related thermal treatment of the unprocessed titanium alloys should be adopted to introduce substantial deformed structures. At the same time, under the suitable hot deformation conditions of titanium alloys, the high dislocation density can significantly increase the mobile dislocation density and also improve the plastic deformation performance [49].

Apart from the control methods of the initial microstructure, the progress of the hot forming process also needs to be proposed to improve the macroscopic plastic formability of titanium alloys. For example, during the hot gas forming of titanium alloys tube, only the tube is heated to the pre-designed elevated temperature within 30 s through the current resistance heating. Such a short heating time effectively inhibits the grain growth and improves the forming performance [2]. A fast-heating method is also suitable for hot stamping of titanium alloy. The microstructure of Ti-6Al-4V alloy after fast heating was in a nonequilibrium state with suppressed grain growth and martensite transformation, which could enhance ductility significantly compared with the slow heating [1].

In addition, the ductility fracture of metals at elevated temperature is closely related to the microstructure evolution, such as DRX and phase transformation. For example, with the softening effect caused by DRX, the local stress concentration is highly relieved, which serves as the driving force of void evolution [18]. Besides, the refined grain size caused by DRX decreases the deformation heterogeneity and the possibility of the occurrence of large voids [19]. Therefore, one can propose a loading path with controllable variable-parameter Z. In the first half of hot deformation with high parameter Z (relatively low deformation temperature and high strain rate), voids are prone to occur, and deformation energy storage is accumulated sufficiently. Before the occurrence of voids, the DRX behaviour is promoted through the decreased parameter Z and the previous accumulated deformation energy storage to improve the forming performance of the component.

## 5. Conclusions

In this paper, an integrated crystal plasticity finite element model of near-α titanium alloys during non-superplastic hot deformation was developed, considering the deformation mechanism of dislocation motion and grain boundary sliding (GBS), the microstructure evolution of dislocation density, dynamic recovery (DRV), dynamic recrystallisation (DRX) and void evolution. The following conclusions were reached:

(1) Based on the actual equiaxed microstructure of a near-α TA15 titanium alloy, the polycrystalline finite element model was established, containing the α phase, β phase and grain boundary (GB) region, in which the GB region was a visualised representation of GBS. A physics-based GBS model was established, in which the normal direction of the motion plane of GB dislocations was the direction vector from the central point of the grain to the points in the GB region.
(2) The quantitative strength ratio between the GB regions and α phase was calculated based on the Zener–Holloman parameter Z and grain size, which determined the subsequent microscopic deformation. With the decrease in parameter Z or grain size, the strength ratio gradually decreased. Moreover, under the certain non-superplastic hot deformation condition, there is always a critical grain size that makes the strength of the GB regions equal to that of the α phase, which increases with the decreased parameter Z.
(3) When the strength of the GB regions is greater than that of the α phase, there are three high strain regions: the β phase region, the intragranular deformation bands through the most favourable slipping (namely, prismatic slipping) and the deformation bands near the GBs through the multiple prismatic, basal and pyramidal-1 slipping. The multiple slipping near the GBs determines the GB misorientations of the following discontinuous DRXed grains, namely [0001] 30°, [$11\bar{2}$0] 75° and [$13\;\bar{8}\;\bar{5}\;3$] 90°.
(4) The dislocation motion accommodating GBS fall into two categories: intragranular dislocation activities and that near the GBs. When the strength of the GB regions is greater than that of the α phase, the activated dislocations pile up inside the grain, which promote the continuous DRX. In turn, the dislocations travel across the grain interior. The average thickness of the accommodating regions near the GBs decreases with the decreasing parameter Z or grain size.
(5) In order to improve the macroscopic plastic formability of difficult-to-form titanium alloys during non-superplastic hot deformation, the control methods of initial microstructure, for example, refining phase/grain size, introducing substantial deformed structures and isothermal hydrogenation treatment can be performed. In addition, fast heating can suppress the grain growth and martensite transformation, and a loading path with controllable variable–parameter Z can suppress damage behaviour by the stimulative DRX behaviour, which all enhance the formability.

## Figures and Tables

**Table 1:** Parameters of crystal plasticity constitutive model of TA15 titanium alloy during non-superplastic hot deformation.

Parameters	Meaning	Value or Expression
μ	shear modulus (GPa)	$\mu_{\alpha ,\beta }=\mu_{0\alpha ,\beta }\left[1+\left(\frac{T-300}{T_{M}}\right)\left(\frac{T_{M}}{\mu_{0\alpha ,\beta }}\frac{d\mu_{\alpha ,\beta }}{dT}\right)\right]$
$C_{ij}$	elastic modulus matrices (GPa)	$C_{ij}=C_{ij0}\frac{\mu \left(T\right)}{\mu_{RT}}$
$f_{\alpha }$	volume fraction of α phase	$f_{\alpha }=0.896\left\{1-exp\left[-0.018\left(1253-T\right)\right]\right\}$
m	strain rate sensitivity exponent	0.05 (700 °C)/0.1 (750 °C)/0.15 (800 °C)
$\rho_{0}$	initial dislocation density (m^−2^)	6×10^14^
$Q_{Act}$	activation energy of deformation (KJ mol^−1^)	459.4 (700 °C)/437.5 (750 °C)/415 (800 °C)
R	gas constant (J K^−1^ mol^−1^)	8.314
b	Burgers vector (10^−10^ m)	2.95 (α, 〈a〉)/5.53 (α, 〈c+a〉)/2.86 (β)
$Q_{GB}$	GB energy per unit area (J m^−2^)	0.43
$Q_{Dis}$	dislocation line energy (J m^−1^)	$1/2\mu b^{2}$
$M_{0}$	reference value of GB mobility (10^−10^ m s^−1^ MPa^−1^)	1.15
ν	Poisson ratio	0.3
δ	a scale factor	0.7
$C_{Void}$	critical value of void nucleation (MPa)	$C_{Void}=4900/lnZ+331.8$
$c_{1}$	fitting constant (s^−m^)	$c_{1}=1.27\times 10^{23}$
$c_{1GBS}$	fitting constant (s^−m^ μm^0.5^)	$c_{1GBS}=1.4\times 10^{22}$
$c_{2}$	fitting constant (MPa)	$c_{2\alpha P}=140$ $c_{2\alpha B}:c_{2\alpha P}:c_{2\alpha Py}:c_{2\alpha Py1}:c_{2\alpha Py2}:c_{2\beta }:c_{2GBS}$ =1:0.7:1:3:3:0.5:1
$c_{3}$	fitting constant	0.1
$c_{4}$	fitting constant	$c_{4}=39.5/lnZ+0.35$
$c_{5}$	fitting constant (m^−1^)	$4.8\times 10^{8}$
$c_{6}$	fitting constant	$c_{6}=0.01\cdot Z^{-1/7.5}$
$c_{7}$	fitting constant (s^−1^)	$c_{7}=4.5\times 10^{-5}\cdot Z^{-1/7.5}$
$c_{8}$	fitting constant (s^m−1^)	$5.5\times 10^{23}$

**Table 2:** Strength relationship of α grain, β phase and GB region of TA15 titanium alloy.

Phase Constitute	$\mathrm{Deformation}\;\mathrm{Strength}\;{\bar{\sigma }}_{\alpha }/{\bar{\sigma }}_{\beta }/{\bar{\sigma }}_{GB}$				
α phase	${\bar{\sigma }}_{\alpha }$				
β phase	${\bar{\sigma }}_{\beta }=0.5{\bar{\sigma }}_{\alpha }$				
GB region	${\bar{\sigma }}_{GB}>{\bar{\sigma }}_{\alpha }$	${\bar{\sigma }}_{GB}={\bar{\sigma }}_{\alpha }$	${\bar{\sigma }}_{\beta }<{\bar{\sigma }}_{GB}<{\bar{\sigma }}_{\alpha }$	${\bar{\sigma }}_{GB}={\bar{\sigma }}_{\beta }$	${\bar{\sigma }}_{GB}<{\bar{\sigma }}_{\beta }$

## Data Availability

The raw/processed data required to reproduce these findings cannot be shared at this time as the data also forms part of an ongoing study.
